# RANKL-Induced Btn2a2 – A T Cell Immunomodulatory Molecule – During Osteoclast Differentiation Fine-Tunes Bone Resorption

**DOI:** 10.3389/fendo.2021.685060

**Published:** 2021-08-04

**Authors:** Michael Frech, Gregor Schuster, Fabian T. Andes, Georg Schett, Mario M. Zaiss, Kerstin Sarter

**Affiliations:** ^1^Department of Internal Medicine 3, Rheumatology and Immunology, Friedrich-Alexander-University Erlangen-Nürnberg (FAU) and Universitätsklinikum Erlangen, Erlangen, Germany; ^2^ Deutsches Zentrum für Immuntherapie (DZI), Friedrich-Alexander-University Erlangen-Nürnberg (FAU) and Universitätsklinikum Erlangen, Erlangen, Germany

**Keywords:** osteoclast (OC), RANKL (receptor activator for nuclear factor k B ligand), bone homeostasis, bone resorption, T cell

## Abstract

Butyrophilins, which are members of the extended B7 family of immunoregulators structurally related to the B7 family, have diverse functions on immune cells as co-stimulatory and co-inhibitory molecules. Despite recent advances in the understanding on butyrophilins’ role on adaptive immune cells during infectious or autoimmune diseases, nothing is known about their role in bone homeostasis. Here, we analyzed the role of one specific butyrophilin, namely Btn2a2, as we have recently shown that Btn2a2 is expressed on the monocyte/macrophage lineage that also gives rise to bone degrading osteoclasts. We found that expression of Btn2a2 on monocytes and pre-osteoclasts is upregulated by the receptor activator of nuclear factor κ-B ligand (RANKL), an essential protein required for osteoclast formation. Interestingly, in Btn2a2-deficient osteoclasts, typical osteoclast marker genes (Nfatc1, cathepsin K, TRAP, and RANK) were downregulated following RANKL stimulation. *In vitro* osteoclast assays resulted in decreased TRAP positive osteoclast numbers in Btn2a2-deficient cells. However, Btn2a2-deficient osteoclasts revealed abnormal fusion processes shown by their increased size. *In vivo* steady state µCT and histological analysis of bone architecture in complete Btn2a2-deficient mice showed differences in bone parameters further highlighting the fine-tuning effect of BTN2a2. Moreover, in rheumatoid arthritis patients and experimental arthritis, we detected significantly decreased serum levels of the secreted soluble Btn2a2 protein. Taken together, we identified the involvement of the immunomodulatory molecule Btn2a2 in osteoclast differentiation with potential future implications in basic and translational osteoimmunology.

## Introduction

Bone loss and musculoskeletal diseases are playing an increasingly important role in medicine (1). Bone is a living organ that is subject to constant change in the form of remodeling processes (2). Bone homeostasis is determined by the activity of osteoclasts (OC) that break down bone matrix and osteoblasts that synthesize bone matrix. Disturbance in this homeostasis can lead to systemic or local bone loss and the development of osteoporosis and bone erosions, respectively (3, 4). Monocyte-colony-stimulating factor (M-CSF) and the receptor activator of NF-κB Ligand (RANKL) are of primary importance for OC differentiation. M-CSF is primarily secreted by osteoblasts (5), and RANKL is mainly synthesized by osteocytes in the context of bone homeostasis, but many other cells such as osteoblasts and T cells can do so as well. Various signal cascades were identified that are triggered by the RANK-RANKL interaction and that induce osteoclast activity and survival such as the inhibitor of NF-kB kinase (IKK), c-jun n-terminal kinase (JNK), p38, or tyrosine kinase SRC (C-Src), Nuclear factor of activated T-cells, cytoplasmic 1 (Nfatc1), the osteoclast-associated receptors (OSCAR) or epidermal growth factor receptors (EGFR) (6–10). Surface receptors that modulate osteoclast activity and regulate such intracellular signaling pathways are of particular interest in bone biology as their manipulation affects bone homeostasis.

Butyrophilins (BTN) comprise a group of transmembrane proteins belonging to the immunoglobulin superfamily. Due to their very similar structure, BTN are seen as members of the B7 protein family. Only 5 proteins of the family have been detected in both humans and mice (11). Members of the protein family are associated with immunological processes such as the activation of B and T cells and are also expressed on immune cells (12–17). Even if the role of BTN proteins in the immune response is not dominant, they could be one of many factors that, e.g. through mutation, that favor the development of inflammatory disease (18–20). The protein Btn2a2 is particularly expressed on thymic epithelial cells and antigen-presenting cells such as dendritic cells, monocytes, and B cells. In recent years, the contribution of Btn2a2 in the context of T-cell activation has been highlighted (13, 21), and in a mouse model with Btn2a2-deficient animals, it was shown that its main function is a negative co-stimulatory effect on T-cell activation (22).

T cells play a special role in osteoclastogenesis, osteoclast activity and recruitment (23). RANKL is expressed on certain T helper cells (Th), Th17 cells, and some of their cytokines, like IL-17, promote osteoclast differentiation (24). In addition, other downstream effector cytokines such as IL-6 and TNF-alpha induce osteoclasts and promote recruitment of osteoclast precursor cells (25). On the other hand, it was shown that regulatory T cells (Treg) can suppress the differentiation of osteoclasts (26) (27). The RANK receptor and its ligand RANKL are also found in various structures of the immune system such as the thymus (28), the lymph nodes (29), or the microfold cells of the intestine (30). Based on the role of T cells in osteoclast differentiation and the effect of Btn2a2 on T cell costimulation we considered to investigate the influence of Btn2a2 on bone homoeostasis. In our data, we can show that Btn2a2 is involved in the differentiation and fusion processes of bone degrading OC.

## Material and Methods

### Mice

Btn2a2^-/-^ mice and wild-type littermates were housed and experiments were conducted under specific pathogen-free conditions and housed in a room at 23 ± 2°C, with 50 ± 10% humidity and a 12hour light/dark. Btn2a2^–/–^ mice were generated by the Wellcome Trust Sanger Institute (Cambridge). Mice were allowed free access to water and regular rodent chow. All of the protocols for animal experiments were approved by the local ethics authorities of the Regierung von Unterfranken.

### Cell Culture

Bone marrow-derived monocytes (BMMs) were isolated and differentiated into Osteoclast precursors (OCP) and mature Osteoclasts (OC) as previously described with modifications indicated (31). Briefly, bone marrow of 8-14-week-old Btn2a2^-/-^ mice and their littermate controls was isolated by flushing femoral and humeral diaphyseal bones. Cells were seeded into 10 cm petri dishes and cultured overnight in complete medium (αMEM, supplemented with 10% L929 conditioned medium, 10% fetal calf serum (FCS) and 1% penicillin/streptomycin). After 24h 2 x 10^5^ cells/well were seeded into 96-well culture plates and grown for 4 days in complete medium containing 50 ng/ml RANKL (R&D Systems, Wiesbaden-Nordenstadt, Germany), unless otherwise stated. Medium was replenished after 72h. Osteoclast differentiation was evaluated by staining fixed cells for TRAP using Leukocyte Acid Phosphatase Kit (Sigma-Aldrich, Taufkirchen, Germany), according to the manufacturer’s instructions.

### Osteoclast Assay

BMMs from Btn2a2^-/-^ and littermate control mice were isolated and differentiated in osteoclast precursors (OCP) and mature osteoclasts (OC) as described previously (31). The TRAP stain of the differentiated and grown OC was performed 5 days after the isolation. Images were taken by Keyence BZ-X710 in 12 sections per well and got joined digitally to create an image of the complete well. Digital measurement technique (Adobe Photoshop^®^) was used to quantify osteoclast growth on *in vitro* images. TRAP-positive, multinucleated OC (with 3 or more nuclei) were designated and counted as OC. The contours of those OC were outlined and the enclosed area was calculated with Adobe Photoshop^®^.

### *In-Vitro* Scratch Assay

For horizontal migration analysis BMMs from Btn2a2^-/-^ and littermate control mice were seeded into 10 cm petri dishes and cultured overnight in complete medium. After 24h, 137.5 × 10^5^ cells/well were seeded into a 35 mm 2-well culture insert (ibidi) in complete medium containing 50 ng/ml RANKL (R&D Systems, Wiesbaden-Nordenstadt, Germany) for additional 12h allowing cells to adhere and spread on the substrate. Subsequently, the culture insert was removed and cells were washed five times with growth medium to remove floating cells. Cells were allowed to migrate back into the scratched area for 24h and counted.

### Bone Resorption Assay

The mineral resorption activity of osteoclasts was conducted using 24-well Corning Osteo Assay Surface Plates (Sigma-Aldrich). BMMs were seeded at a density of 8 × 105 cells/well in complete medium supplemented with RANKL (50 ng/ml) and cultured for 5 days, with medium exchange after 96h. After removing cells with deionized water, resorption pit formation was visualized by von Kossa staining and the resorptive area was quantified using ImageJ (National Institutes of Health, Bethesda, MD, USA).

### RT-PCR Analysis

Samples were lysed with TriFast™ (Peqlab) and RNA was isolated following the instructions of the manufacturer. cDNA was generated using the High Capacity cDNA Reverse Transcription Kit (Applied Biosystems™) and analyzed using SYBR^®^ Select Master Mix (Thermo Fisher Scientific) on a QuantStudio™ 6 Flex Real-Time PCR Instrument (Thermo Fisher Scientific). Gene expression results were expressed as arbitrary units relative to expression of the house keeping gene Glycerinaldehyd-3-phosphat-Dehydrogenase (GAPDH), unless indicated otherwise. Primer sequences are as follows; 

GAPDH: 5’-GGGTGTGAACCACGAGAAAT-3’ and 5’-CCTTCCACAATGCCAAAGTT-3’,

Btn2a2: 5’-ATGACCAGGCAACCATGAAGC-3’ and 5’-TCATAGGGGTCTCTCCACA-3’,

RANK: 5’-GCCCAGTCTCATCGTTCTGC-3’ and 5’-GCAAGCATCATTGACCCAATTC-3’,

OPN: 5’-TCCTTAGACTCACCGCTCTT-3’ and 5’-TCTCCTTGCGCCACAGAATG-3’,

Cathepsin K: 5’-AGGGCCAACTCAAGAAGAAAACT-3’ and 5’-TGCCATAGCCCACCACCAACACT-3’,

TRAP: 5’-GGCCGGCCACTACCCCATCT-3’ and 5’-CACCGTAGCCACAAGCAGGACTCT-3’,

C-SRC: 5’-CGTGGCTGTCACCAAGGACCC-3’ and 5’-TGGTGCTTTCCCGCACGAGG-3’,

Nfatc1: 5’-CGGCGCAAGTACAGTCTCAATGGCG-3’ and 5’-GGATGGTGTGGGTGAGTGGT-3’, 

ATP6v0d2: 5’-TCAGATCTCTTCAAGGCTGTGCTG-3’ and 5’-GTGCCAAATGAGTTCAGAGTGATG-3’, 

Dcstamp: 5’-TTTGCCGCTGTGGACTATCTGC-3’ and 5’-GCAGAATCATGGACGACTCCTTG-3’, 

Ocstamp: 5’-TTGCTCCTGTCCTACAGTGC3-3’ and 5’-GCCCTCAGTAACACAGCTCA-3’, 

Csf1r: 5’-TGGATGCCTGTGAATGGCTCTG-3’ and 5’-GTGGGTGTCATTCCAAACCTGC-3’, 

Cd44: 5’-TGGATCCGAATTAGCTGGAC-3’ and 5’-AGCTTTTTCTTCTGCCCACA-3’,

cfos: 5’-CTCTGGGAAGCCAAGGTC-3’ and 5’-CGAAGGGAACGGAATAAG-3’.

### Transcriptome Analysis of Osteoblasts

For transcriptome profiling of primary osteoblasts public RNA-seq data (32) was obtained from the sequence read archive (SRA, BioProject accession: PRJNA648106). Transcript-level abundances were quantified using Salmon (v1.3.0) (33) using the reference transcriptome GRCm39 from ensemble database. Differential expression analysis was performed with DESeq2 (v1.28.1) (34) with default parameters.

### Micro-Computed Tomography Analysis (µCT)

For μCT imaging, 12-week old male Btn2a2^-/-^ and littermate controls were used. µCT imaging was performed using the cone-beam Desktop Micro Computer Tomograph “μCT 40” (SCANCO Medical AG, Bruettisellen, Switzerland). The settings were optimized for calcified tissue visualization at 55 kVp with a current of 145 μA and 200 ms integration time for 500 projections/180°. For the segmentation of 3D-Volumes, an isotropic voxel size of 8.4 μm and an evaluation script with adjusted greyscale thresholds of the operating system “Open VMS” (SCANCO Medical) were used.

### Histology

For histological analysis, tibial bones were fixed in Histofix (Roth) for 12h and decalcified in EDTA (Sigma-Aldrich). Serial paraffin sections (2 μm) were stained for H&E and tartrate resistant acid phosphatase (TRAP) using a Leukocyte Acid Phosphatase Kit (Sigma) according as to the manufacturer’s instructions. Undecalcified bones were embedded in methacrylate and 5µm section were cut. Toluidine blue staining was performed for quantification of osteoblasts. Osteoclast and osteoblast numbers were quantified using a microscope (Nikon) equipped with a digital camera and an image analysis system for performing histomorphometry (Osteomeasure; OsteoMetrics).

### Flow Cytometry

Single-cell suspensions of whole bone marrow were incubated on ice with conjugated antibodies in PBS containing 2% FCS and 5 mM EDTA (Merck). Dead cells were excluded with Fixable Aqua Dead Cell Stain (Thermo Fisher Scientific). For detection of the progenitor populations granulocyte-macrophage progenitors (GMP), common myeloid progenitors (CMP) and megakaryocyte erythrocyte progenitors (MEP), cells were stained with fluorochrome-conjugated anti-lineage (CD3, clone 17A2; Ly-6G, clone M1/70; CD11b, clone RB6-8C5; CD45R(B220), clone RA3-6B2; Ter-119, clone Ter-119, cat. #133311, Biolegend), anti-CD117 (clone 2B8, Biolegend), anti-CD127 (clone A7R34, Biolegend), anti-Ly6A/E (clone D7, Biolegend), anti-CD16/32 (clone 93, Biolegend) and anti-CD34 (clone RAM34, BD). Flow cytometry analysis was performed on the cytoflex platform (Beckman Coulter) and analyzed with FLowJo v.10 software (TreeStar).

### Measurement of Serum Cytokines and Serum Btn2a2

Serum levels of osteocalcin (Nordic Bioscience), C-terminal telopeptide α1 chain of type I collagen (CTX-I) (RatLaps; Nordic Bioscience), and Btn2a2 (MyBioSource) were measured by enzyme-linked immunosorbent assay according to the manufacturer’s instructions.

### Collagen-Induced Arthritis (CIA)

CIA was induced in 8-week-old female DBA/1J mice by s.c. injection of 100 μl containing 0,25 mg chicken type II collagen (CII; Chondrex, Redmond, WA) in complete Freund adjuvant (CFA; Difco Laboratory, Detroit, MI), and 5 mg/mL killed Mycobacterium tuberculosis (H37Ra) at the base of the tail. Mice were re-challenged after 21 days by intradermal immunization at the base of the tail with this emulsion. Serum samples were collected at day 30 post injection.

### Statistical Analysis

Data are expressed as mean ± s.e.m. unless otherwise indicated in the figure legends. Analysis was performed using Student’s t-test, single comparison, or analysis of variance (ANOVA) test for multiple comparisons (one-way or two-way ANOVA followed by Tukey’s or Bonferroni’s multiple comparisons test, respectively). All experiments were conducted at least two times. P-values smaller than 0.05 were considered significant and are shown as p<0.05 (*), p<0.01 (**) p<0.001 (***), or p<0.0001 (****). Graph generation and statistical analyses were performed using the Prism version 8 software (GraphPad, La Jolla, CA) or R.

## Results

### Btn2a2 Expression Is Up Regulated During Osteoclast Formation

**Figure 1:**
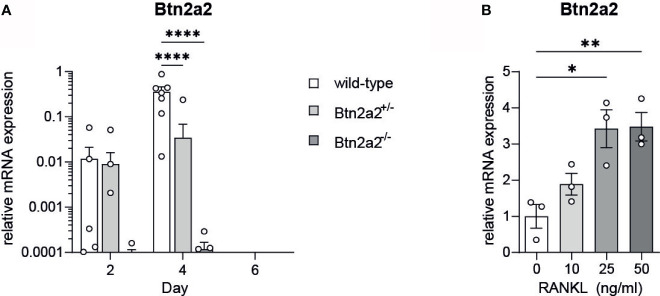
Btn2a2 is regulated during osteoclastogenesis in a RANKL-dependent manner. **(A)** Btn2a2 expression was analyzed by quantitative RT-PCR at days 2, 4 and 6 during osteoclast assay from Btn2a2-/- , Btn2a2+/-, and wild-type littermate controls. Relative expression values normalized to the housekeeping gene GAPDH are shown. **(B)** Quantita­ tive RT-PCR for determination of relative mRNA levels of Btn2a2 normalized on GAPDH, after 2 hours stimulation of osteoclast precursors with indicated RANKL concentrations. Data are representative of 2 independent experiments, with 3 mice per group. Significance was assessed using one- **(B)** or two-way ANOVA **(A)** and Bonferroni's post-hoc test. Data are shown as means± SEM. *P < 0.05; **P < 0.01. ****P < 0.0001.

To test our hypothesis of a connection between Btn2a2 expression and osteoclast (OC) differentiation, we analyzed the expression of Btn2a2 in osteoclast progenitor cells (OCP) after addition of RANKL. We observed that expression of Btn2a2 in OCP from wild-type mice is upregulated during the process of osteoclastogenesis, with a peak 4 days after stimulation with RANKL and M-CSF to OCP (**Figure 1A**). No expression of Btn2a2 was detected in OC from Btn2a2^-/-^ mice 2, 4, and 6 days after RANKL addition. At day 2 Btn2a2 expression in wild-type and in mice heterozygous for Btn2a2 (Btn2a2^+/-^) was low and increased at day 4 with an intermediate Btn2a2 expression detected in Btn2a2^+/-^ mice, while at 6 days after RANKL addition expression of Btn2a2 could not be detected (**Figure 1A**). Interestingly we found that the up-regulation of Btn2a2 expression occurs in a dose-dependent manner with a plateau being reached with the addition of 25 ng/ml RANKL (**Figure 1B**), thus proving that Btn2a2 expression is dependent on RANKL stimulation and thereby indicating that it is involved in the process of OC differentiation. Analyzing published data from primary osteoblast during differentiation (32) revealed no expression of Btn2a2 (**Supplementary Figures 2B, C** **)**, indicating that the role of Btn2a2 in bone homeostasis is restricted to effects on osteoclasts.

### Loss of Btn2a2 Reduces Expression of Marker Genes Driving OC Formation

**Figure 2:**
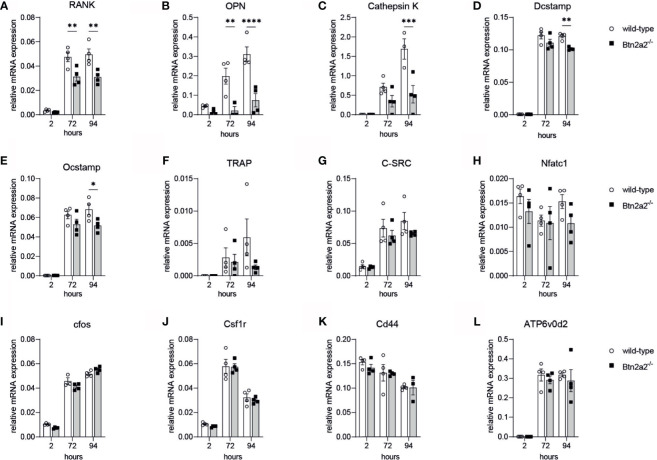
Btn2a2-/- Osteoclasis reveal altered expression of OC-specific transcriptional signature during osteoclastogenesis. **(A–L)** Expression of osteoclast-related genes in wild-type and Btn2a2' osteoclasts after stimulation of osteoclast precursors with RANKL (50 ng/ml)for the indicated time points. Relative expression values normalized to the housekeeping gene GAPDH are shown. Data are representative of two Independent experiments, with 3 mice per group. Significance was assessed using two-way ANOVAand Bonferroni's post-hoc test. Data are shown as means :t SEM. *P < 0.05; **P < 0.01; ***P < 0.001; ****p < 0.0001.

To further investigate the role of Btn2a2 in the osteoclast differentiation, we analyzed the expression of markers involved in OC proliferation, differentiation, activation and fusion in OC derived from Btn2a2^-/-^ mice compared to wild-type littermate controls. We observed that lack of Btn2a2 leads to significantly different expression of analyzed markers of OC during osteoclastogenesis (**Figure 2**). 72h and 94h following RANKL stimulation. RANK and OPN expression of Btn2a2^-/-^ OC were significantly lower than expression measured in wild-type OC (**Figures 2A, B** **)**. Cathepsin K expression was significantly decreased in Btn2a2^-/-^ OC after 94h (**Figure 2C**), as well as DC-Stamp and OC-Stamp expression (**Figures 2D, E**). TRAP expression was reduced as well though not significantly in Btn2a2^-/-^ OC compared to wild-type OC (**Figure 2F**), while c-Src, NFATc1, cfos, Csf1r, CD44, and ATPv0d2 expression remained unchanged (**Figures 2G–L**). These data further underline the connection between bone homeostasis and Btn2a2 expression in OC.

### Loss of Btn2a2 Leads to a Reduced Number of Bone Degrading Osteoclasts

**Figure 3:**
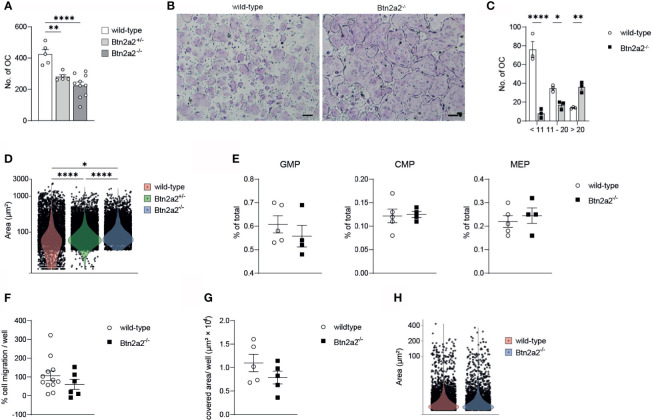
Loss of Btn22 leds to reduced numbers of bone degrading osteoclasts. **(A)** quantification of the number of multinucleated TRAP+ osteoclasts from Wild-type, Btn2a2+/- and Btn2a2-/-mice after osteoclast assay. **(B)** Trap staining of Wild-type and Btn2a2-/- osteoclasts at day 5 of osteoclast assay and **(C)** quantification of the number of multinucleated TRAP+ osteoclasts by their number of nuclei. **(D)** violin plots displaying area of multinucleated TRAP+ osteoclasts at day 5 of osteoclast assay of wild-type Btn2a2+/- and Btn2a2-/- mice with each dot representing one osteoclast. **(E)** Flow cytometric analysis of bone marrow progeni­tor cells of wild-type and Btn2a2-/- mice. **(F)** OCP migration abilityof wild-type and Btn2a2-/- OCPs was assessed in vitro. Quantification of migrated cells was performed with images captured after 24 hours. Data are representative of two independent experiments, with 3 mice per group. **(G)** Analysis of resorption acitvity of Btn2a2-/- and their wild-type liltermates after 5 days of culture. **(H)** Resorbed area per osteoclast. Data are representative of two independent experiments, with 3-4 mice per group **(B, C, E–G)** or represent two pooled experiments **(A, D, H)**. Significance was assessed using one-way **(A)** or two-way ANOVA **(C)** and Bonferro­ni's post-hoc test or using two-tailed Students t-test. **(D)** Signifiance was assessed using Krusaii-Wallis test and pairwise comparisons using Wilcoxon rank sum test with Bonferroni's p value adjustments. **(B)** Scale bar indicates 200 μm. Data are shown as means t SEM. *P < 0.05; **P < 0.01; ****P < 0.0001.

Since we observed a clear difference in expression of differentiation and activation markers of Btn2a2^-/-^ OC compared to wild-type OC, we investigated if these changes in OC marker gene expression is relevant for the differentiation of OC. When analyzing OC differentiation assays we observed that the overall number of OC formed of progenitor cells after addition of M-CSF and RANKL was significantly reduced in Btn2a2^-/-^ mice, with an intermediate number of OC formed from Btn2a2^+/-^ OCP (**Figure 3A**) thereby reflecting the results of decreased expression of OC marker genes relevant for OC formation. Interestingly, while displaying overall less OC in the assays with Btn2a2^-/-^ OCP, we observed significantly higher numbers of OC with more than 20 nuclei in Btn2a2^-/-^ compared to wild-type OC with an intermediate phenotype in Btn2a2^+/-^ OC (**Figures 3B–D**). These data clearly indicate that Btn2a2 favors formation of OC *in vitro* and that loss of Btn2a2 also leads to an abnormal fusion process. In contrast, we did not observe abnormalities in the BM compartment of Btn2a2^-/-^ mice compared to their wild-type counterparts as analyzed by flow cytometry for the frequencies of granulocyte-macrophage progenitors (GMP), common myeloid progenitors (CMP) and megakaryocyte erythrocyte progenitors (MEP) (**Figure 3E**). In addition, no defects in migratory behavior of Btn2a2^-/-^ OC compared to wild-type OC was detected (**Figure 3F**) as well as no difference in *in vitro* resorption capacity (**Figure 3G**) and no differences in resorbed surface per OC was observed (**Figure 3H**).

### Loss of Btn2a2 Changes Trabecular Bone Architecture *In Vivo*

**Figure 4:**
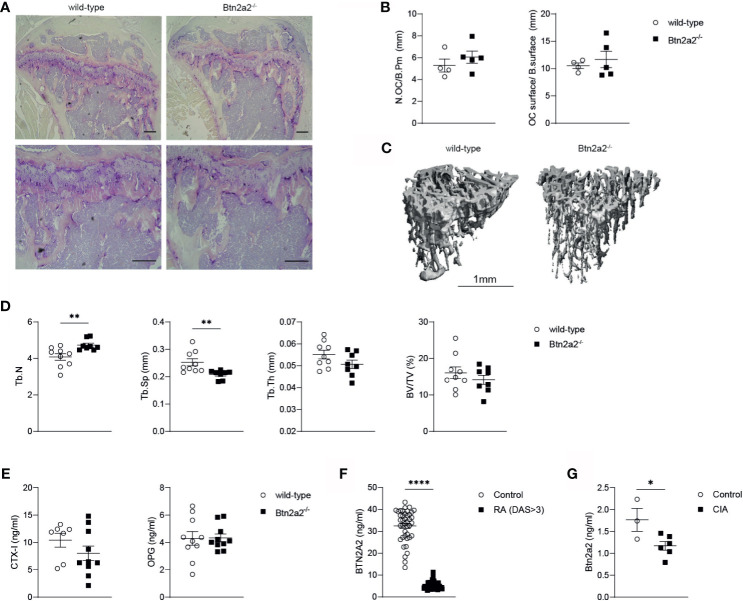
Loss of Btn2a2 changes bone architecture *in vivo*. **(A)** Representative Images of tibiae with TRAP staining of male Btn2a2-/- mice compared with their wild type littermates at 12 weeks of age. Scale bar indicates 200 μm. **(B)** Histomorphometric analysis of the number of osteoclasts per bone perimeter (N.OC/B.Pm) and the osteoclast surface per bone surface (OC.surtace/B.surtace). **(C)** μCT images of the skeletal phenotype of male Btn2a2-/- mice and their wild-type littermates at 12 weeks of age. Scale bar indicates 1 mm. **(D)** μCT analysis of trabecular bone parameters of tibial bone including bone volume to trabecular volume (BV/TV), trabecular thickness (Tb.Th.), trabecular number (Tb number), and trabecular separation (Tb.Sp.). **(E)** Serum levels of CTX-1 and OPG from Btn2a2-/- mice and wild-type littermates at 12 weeks of age were measured by ELISA. **(F)** BTN2A2 serum levels from RA patients and healthy controls. **(G)** Btn2a2 serum levels from CIA mice compared to healthy controls at day 30 of CIA. Significance was assessed using Student's t-test. Date are shown as means ± SEM. *P < 0.05; **P < 0.01; ****p < 0.0001.

Since Btn2a2 is up-regulated in OC after RANKL addition and Btn2a2^-/-^ OC displayed altered expression of differentiation and activation markers that lead to a reduced OC formation and abnormal fusion process, we further investigated if the loss of Btn2a2 has an effect on bone homeostasis *in vivo*. Therefore, we analyzed bones from wild-type and Btn2a2^-/-^ mice by histology and µCT for differences in bone parameters. Histological analysis of bones from W and Btn2a2^-/-^ mice showed no differences both in numbers of OC per bone perimeter and in OC surface per bone surface (**Figures 4A, B** **)**. We also did not observe any differences between Btn2a2^-/-^ and wild-type mice in numbers of osteoblasts (**Supplementary Figure 2A**). However, results obtained from µCT analysis of tibial bones from Btn2a2^-/-^ mice showed a significant increase in trabecular number and a significant decrease in trabecular separation when compared to tibia from wild-type mice, whereas bone volume was not statistically different in Btn2a2^-/-^ mice (**Figures 4C, D** **)**. This indicates that Btn2a2 may subtly influence the remodeling process of bone homeostasis *in vivo*. In addition to analyzing bone parameters, we also measured serum levels of C-terminal telopeptide of type 1 collagen (CTX) and osteoprotegerin (OPG) as markers of bone homeostasis in wild-type and in Btn2a2^-/-^ mice. However, CTX and OPG serum levels were unchanged (**Figure 4E** **)**. Another indication that Btn2a2 could play a role in bone homeostasis comes from measurements of Btn2a2 levels in sera of RA patients as well as from mice with a collagen-induced arthritis (CIA), in which the levels of Btn2a2 were significantly reduced in RA patients and CIA mice compared to normal healthy controls (**Figures 4F, G** **)**, indicating that Btn2a2 is involved in diseases in which bone homeostasis is affected.

## Discussion

Osteoclast (OC) differentiation and activation is a multistep process that is critically regulated by RANKL (35, 36). Although RANKL is the main driver of OC differentiation, a number of additional factors have been revealed to be necessary for osteoclastogenesis and a multitude of hormones and cytokines modulate osteoclast formation (37–41). Herein, we show that RANKL induces the expression of the co-stimulatory molecule Btn2a2 in OC. To our knowledge, this is the first observation that Btn2a2 is expressed in OC upon RANKL stimulation and its dose dependent increase in expression indicates that it is involved in the process of OC formation. OC formation includes a series of regulatory steps, including differentiation, activation, migration, as well as their fusion into mature multinucleated OCs that subsequently initiate osteoclastic bone resorption (42, 43). To further investigate the role of Btn2a2 in these processes, we made use of a loss of function model for Btn2a2 and analyzed expression of known, essential marker genes involved in OC formation. We observed that OC deficient for Btn2a2^-/-^ showed decreased expression in RANK, OPN, and cathepsin K, DC-Stamp, and OC-Stamp. RANK is critical for the OC formation and activation, it binds RANKL thereby activating signaling pathways in OC including the members of the TNF receptor activating factor (TRAF) family (44). OPN is one of the most abundant non-collagenous proteins in bone and is an important regulator of inflammation and biomineralization and regulates bone cell adhesion, osteoclast function, and matrix mineralization (45). However, mice lacking OPN show normal development and bone structure but display altered OC formation *in vitro* (46).TRAP is able to degrade skeletal phosphoproteins and is secreted by the OC during bone resorption and secretion correlates with resorptive behavior (47, 48). Cathepsin K is the major mediator of osteoclastic bone resorption (49). Upon differentiation and fusion, OC produce increasing amounts of cathepsin K, and this appears to be regulated by RANKL (50). Down-regulation of these marker genes in Btn2a2^-/-^ progenitor cells suggests a functional defect of OC, further underlining a role of this molecule in the process of OC formation. And indeed, when analyzing OC formation assays, we observed that in those assays in which myeloid progenitors from Btn2a2^-/-^ mice were stimulated with M-CSF and RANKL, significantly less OC were formed compared to assays using wild-type myeloid progenitors. With reduced Cathepsin K expression in Btn2a2^-/-^ OC, we would expect less bone resorption by Btn2a2^-/-^ OC compared to wild-type OC. However, we did not observe difference in resorption capacity between wild-type and Btn2a2^-/-^ OC. This discrepancy could be due to the formation of giant OC in Btn2a2^-/-^, which may compensate the overall lower capacity of resorption. Moreover, although Btn2a2^-/-^ OC seem to be bigger, we observed in total more OC in wild-type compared to Btn2a2^-/-^ mice, which also explains an unchanged BV/TV between wild-type and Btn2a2^-/-^ conditions.

Interestingly, when using Btn2a2^-/-^ monocytes, the number of very large OC (more than 20 nuclei) was significantly increased in comparison to wild-type conditions, pointing to an abnormal fusion process of OC in Btn2a2^-/-^ mice. Since we observed giant OC in Btn2a2^-/-^, we analyzed fusion markers of OC to detect a possible defect in the fusion process. We observed significantly reduced expression of DC-Stamp and OC-Stamp in Btn2a2^-/-^ OC, which seems contradictory to an expected enhanced fusion rate of Btn2a2^-/-^ OC. This may be explained by other proteins that play a role in fusion. Little is known about OC fusion, which is a property of mature osteoclasts and is required for osteoclasts to resorb bone. A number of molecular mediators were reported to be important for osteoclast fusion (51–55). Nevertheless, the complete sequence of events leading from stimulation of osteoclastogenesis by RANKL to formation of large osteoclasts capable of bone destruction is incompletely understood. In a recent study, it has been shown that OC fission into osteomorphs and can recycle back into OC during RANKL-stimulated bone resorption (56), and since loss of Btn2a2 leads to reduced RANK expression, a decrease in RANK-RANKL signaling could lead to a reduction of recycling and fission events and therefore to the development of bigger OC. More research needs to be done on investigating the role of Btn2a2 in the fusion events of OC and it could be that recycling from osteomorphs is not possible in Btn2a2^-/-^ OC (56, 57). In order to reveal the role of Btn2a2 in the fusion process of OC, further investigations need to be done, by analyzing expression levels of known fusion genes and the process of fission into osteomorphs and fusion back to OC in Btn2a2^-/-^.

*In vivo*, Btn2a2^-/-^ mice showed differences in trabecular architecture with significantly reduced separation of trabecula and increased trabecula numbers but no differences in bone volume per total volume were observed. The loss of Btn2a2 in the complete knockout mice therefore resulted in an altered bone architecture without a change in total bone mass. From the clear effects in OC from wild-type and Btn2a2^-/-^ mice *in vitro* one would expect that in the Btn2a2^-/-^ mice there is less bone degradation due to impaired OC differentiation, however, the formation of giant OC in Btn2a2^-/-^ cells may counteract this effect *in vivo*, since these giant OC may be able to absorb more bone due to the increased bone surface they can cover.

Moreover, since the work presented here has been conducted in complete Btn2a2^-/-^ mice, other effects of Btn2a2 may have masked the effects on bone homeostasis. Both resorption and formation of bone are tightly coupled processes, and the role of Btn2a2 on other cells needs to be taken into account. A possible role of Bn2a2 on osteoblast function is not known at present. In addition to osteoblasts, T and B lymphocytes are also known to influence the formation and activation of OC.

T cells express RANKL and thereby activated T cells may induce osteoclastogenesis (58), however major T cell cytokines such as IFNg, IL-4 and IL-10 are inhibitory to *in vitro* osteoclastogenesis (23, 59). Regulatory T cells (Treg) have been shown to suppress OC formation and protect from local and systemic bone destruction in arthritis (27, 60, 61). Btn2a2 binds to activated T cells and inhibits activation (13), and Btn2a2 induces Foxp3 expression and a regulatory phenotype in T Lymphocytes (21). As mentioned, loss of Btn2a2 results in a skewed T cell response towards a reduced Treg response (22). Activated T lymphocytes supporting osteoclast formation (58) and reduced Treg numbers in Btn2a2^-/-^ may have an influence on OC differentiation and function *in vivo*. Thus, the effects of T cells on the formation of OC is dependent on a balance between their positive and negative factors. Also other cell types than osteoblasts and T- and B lymphocytes influence OC formation. It has been shown, that ILC2 can suppress OC formation (62, 63) and in our own unpublished work we detected Btn2a2 expression in ILC2 with significant immunomodulatory consequences.

In summary, our data point to a role of Btn2a2 in the differentiation of OC as well as its fusion process highlighting the role of T cell molecules on bone homeostasis.

## Data Availability Statement

The datasets presented in this study can be found in online repositories. The names of the repository/repositories and accession number(s) can be found in the article/**Supplementary Material**.

## Ethics Statement

The studies involving human participants were reviewed and approved by Ethikkommittee der FAU. The patients/participants provided their written informed consent to participate in this study. The animal study was reviewed and approved by Regierung von Unterfranken.

## Author Contributions

Study design, GeS, MZ, and KS. Study conduct, MF and GrS. Data collection, MF, GrS, FA, MZ, and KS. Data analysis, MF, GrS, and KS. Data interpretation, MF, GrS, MZ, and KS. Drafting manuscript, MF, MZ, and KS. Revising manuscript content, GeS, MZ, and KS. Approving final version of manuscript, MZ and KS. KS takes responsibility for the integrity of the data analysis. All authors contributed to the article and approved the submitted version.

## Funding

This work was supported by the Deutsche Forschungsgemeinschaft (DFG) DFG-SPP1937 KS and MZ, by the Dr. Rolf Schwiete Stiftung for MF, KS, MZ and by the Else Kröner-Fresenius Stiftung MF and KS. Additional funding was received by the Interdisciplinary Centre for Clinical Research, Erlangen (IZKF) for KS.

## Conflict of Interest

The authors declare that the research was conducted in the absence of any commercial or financial relationships that could be construed as a potential conflict of interest.

## Publisher’s Note

All claims expressed in this article are solely those of the authors and do not necessarily represent those of their affiliated organizations, or those of the publisher, the editors and the reviewers. Any product that may be evaluated in this article, or claim that may be made by its manufacturer, is not guaranteed or endorsed by the publisher.
