# Supplementary figures and images for: RANKL-Induced Btn2a2 – A T Cell Immunomodulatory Molecule – During Osteoclast Differentiation Fine-Tunes Bone Resorption
Source: Front Endocrinol (Lausanne). 2021 Aug 4;12:685060. doi: 10.3389/fendo.2021.685060 (PMC8371446; doi:10.3389/fendo.2021.685060)

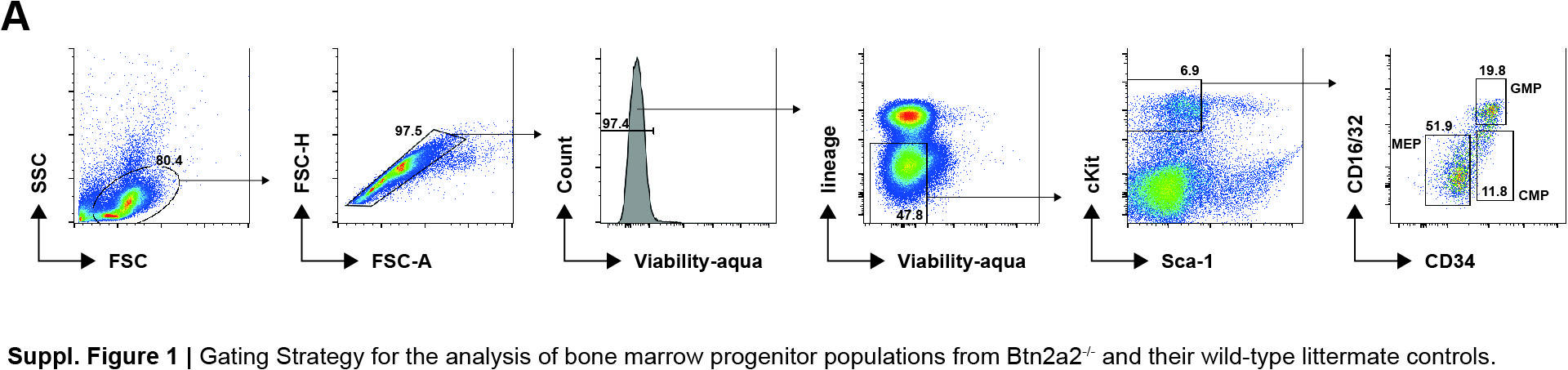

Supplement: Supplementary file 1 [file Image_1.jpeg]

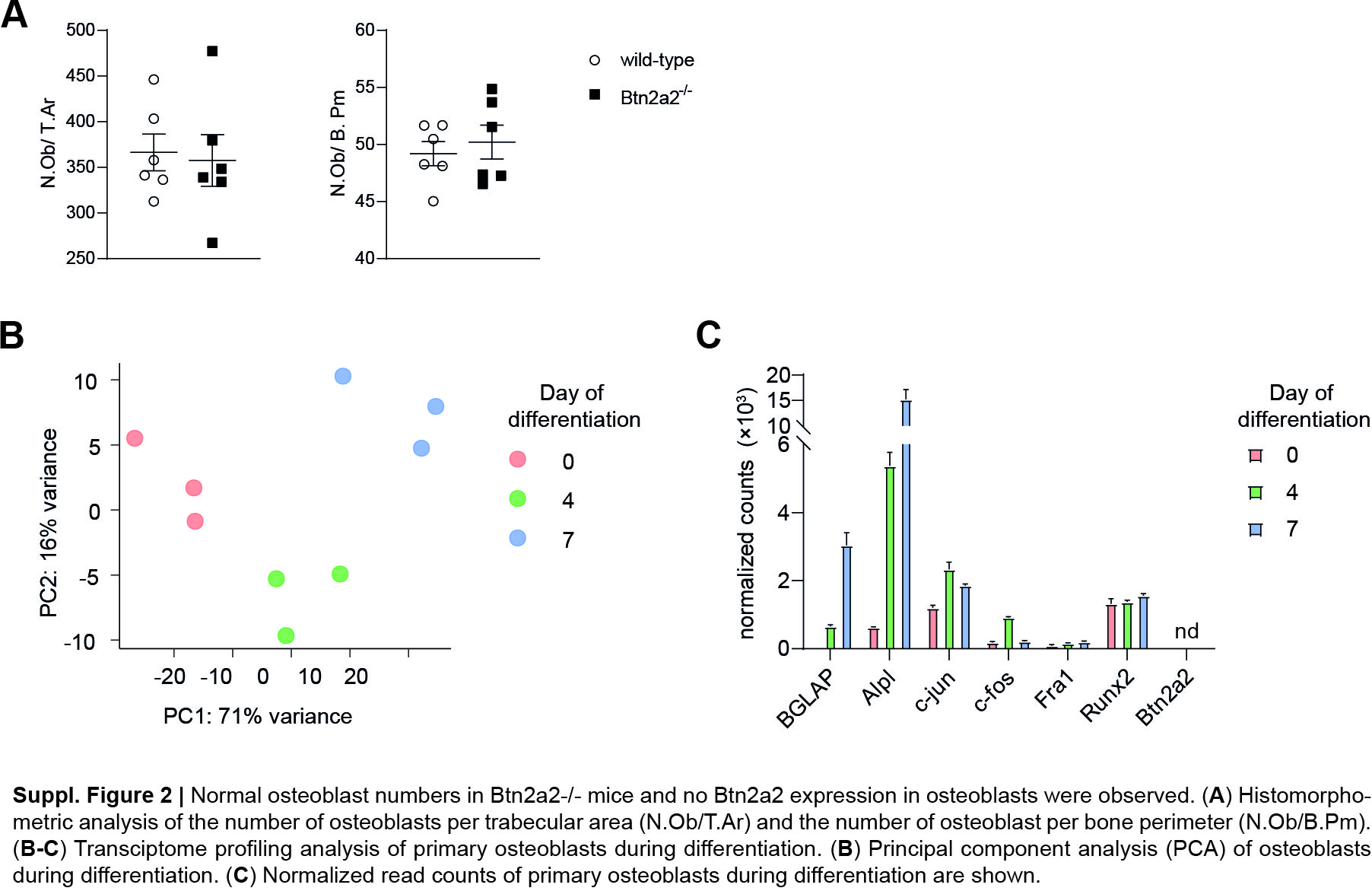

Supplement: Supplementary file 2 [file Image_2.jpeg]
